# Loss of the Orphan GPCR *srd-44* Does Not Alter Chemotaxis of *Caenorhabditis elegans* to Carolina Mantle Slug Mucus

**DOI:** 10.17912/micropub.biology.002287

**Published:** 2026-10-03

**Authors:** Jay Garaycochea, Milo Roth

**Affiliations:** 1 Biological Sciences, Goucher College, Towson, MD, US

## Abstract

*C. elegans* detect volatile odorants through amphid sensory neurons that express diverse G protein-coupled receptors (GPCRs), many of which remain functionally uncharacterized. We evaluated the candidate chemosensory GPCR *srd-44* using the RB1526 mutant strain in chemotaxis assays against diacetyl and Carolina mantle ( *Philomycus carolinianus* ) slug mucus. Wild-type and mutant animals exhibited similar chemotaxis responses to both stimuli across a range of concentrations. Under these conditions, these results do not support a role for *srd-44* in chemotaxis toward diacetyl or Carolina mantle slug mucus. These findings contribute to the functional evaluation of an orphan chemosensory GPCR in *C. elegans* .

**Figure 1. Loss of srd-44 is not associated with reduced chemotaxis to various odorants:** A. Chemotaxis index (CI) of wild-type ( N2 ) and RB1526 ( *srd-44 ( ok1831 ))* animals toward diacetyl (1:100 v/v in ethanol), Carolina mantle slug mucus extract (10 ^0^ dilution), Isoamyl Alcohol (1:100 v/v in ethanol), 1-Octanol (1:10 v/v in ethanol), and ethanol control after 60 minutes. CI was calculated as (T − C) / (T + C), where T represents animals in the test quadrants and C represents animals in ethanol control quadrants. Data are shown as individual biological replicates with mean ± SD. Statistical analysis was performed using two-way ANOVA (genotype x stimulus) followed by Tukey's multiple comparisons test. B. CI of wild-type and RB1526 animals towards diacetyl concentrations ranging from 1.14 × 10⁻⁵ M to 1.14 M after 60 minutes. Data shown as mean ± SD from 3-10 biological replicates per concentration. C. CI of wild-type and RB1526 animals towards Carolina Mantle Slug mucus concentrations ranging from a 10 ^0^ to 10 ^-5^ dilution from original sample after 60 minutes. Data shown as mean ± SD from 3-4 biological replicates per concentration. No consistent differences in chemotaxis responses were observed between wild-type and RB1526 animals across the concentration range tested.

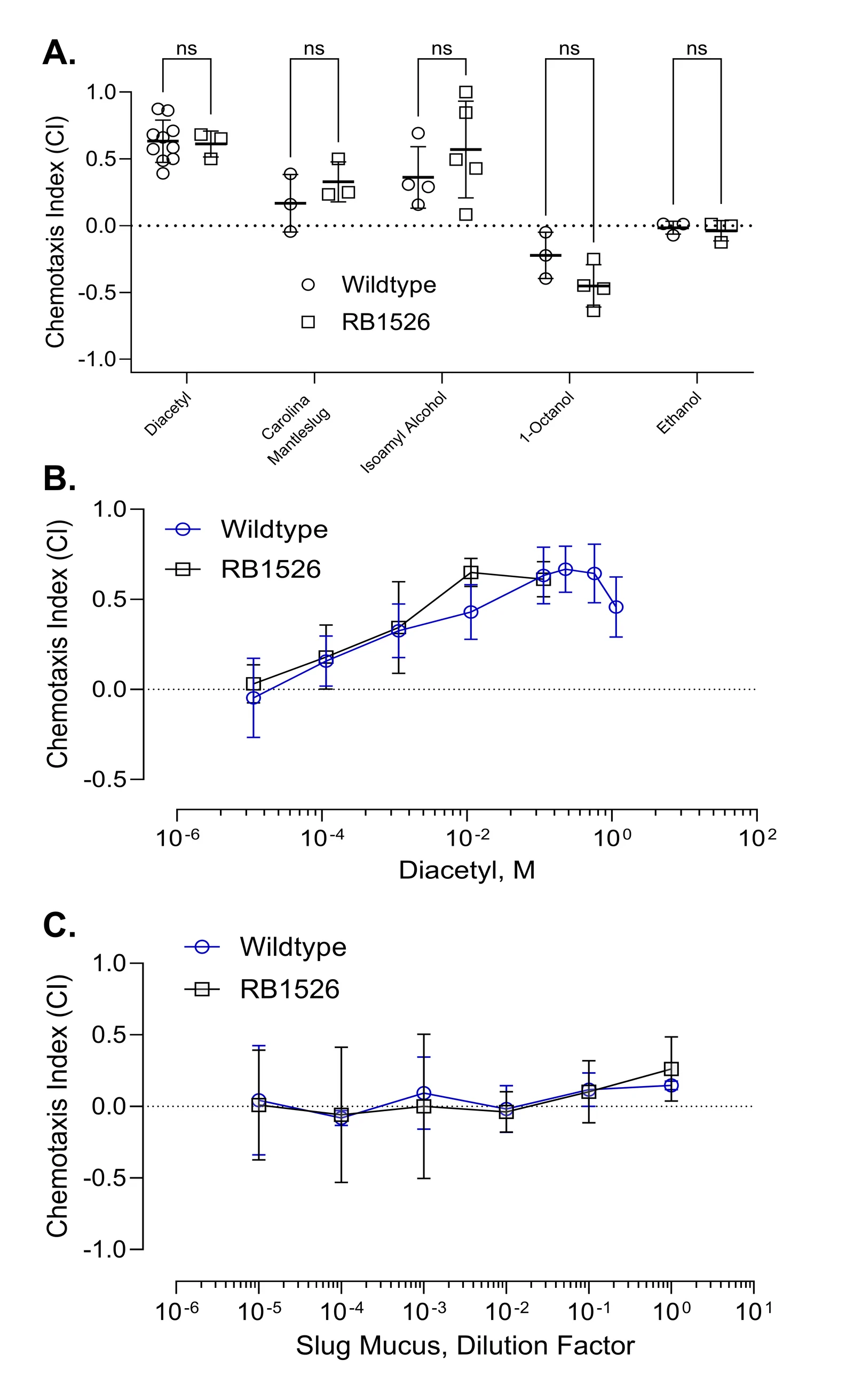



## Description

*C. elegans* detects volatile odorants through amphid sensory neurons that express diverse G protein-coupled receptors (GPCRs), many of which remain functionally uncharacterized (Bargmann et al., 1993; Robertson & Thomas, 2006). Members of this large chemosensory GPCR family mediate responses to both defined odorants and complex biological compounds (Bargmann et al., 1993; Kim et al., 2009a; Troemel et al., 1995a). Identifying the ligands of orphaned GPCRs is a challenging and time-consuming task that requires a comprehensive approach integrating a multitude of techniques to understand the full range of GPCR responses (Jobe & Vijayan, 2024). One potential approach is using biological complex compounds with gas chromatography/mass spectrometry to screen for candidate metabolites (Gaerlan et al., 2025).

*C. elegans* coexist in natural environments with terrestrial invertebrates including isopods and gastropods such as slugs (Frézal & Félix, 2015). Slug mucus contains a mixture of proteins, glycoproteins, and small metabolites that act as volatile compounds and may serve as multimodal chemical cues (Ballard et al., 2021; Smith & Morin, 2002). Members of the large chemosensory GPCR family expressed in amphid neurons mediate responses to both defined odorants and complex biological compounds (Bargmann et al., 1993; Kim et al., 2009b; Troemel et al., 1995b). The properties of slug mucus position it as a candidate for screening odorant GPCRs in *C. elegans* .

*srd-44* encodes a predicted seven-transmembrane receptor belonging to the serpentine receptor class delta (srd) family, a group of genes enriched in chemosensory neurons (Robertson & Thomas, 2006; Troemel et al., 1995a; Vidal et al., 2018). A previous reporter-based study suggested expression of *srd-44* in ASH amphid sensory neurons, although endogenous expression patterns remain incompletely defined (Wood & Ferkey, 2019). ASH neurons are canonically implicated in avoidance responses to nose touch and avoidance of noxious chemicals (Kaplan & Horvitz, 1993); however, the valence of chemosensory responses in C. elegans is not strictly fixed by neuron identity and can shift depending on stimulus concentration, context, and the specific receptor(s) engaged (Khan et al., 2022). Despite its predicted classification as a chemosensory receptor, no ligand or defined behavioral function has been reported for srd-44 . Given its putative expression in ASH sensory neurons, srd-44 was selected as a candidate for behavioral screening to test its role in mediating responses to a panel of attractive and aversive chemosensory stimuli.

Wild-type ( N2 ) and RB1526 ( *srd-44 ( ok1831 )) C. elegans* were compared for chemotaxis towards the volatile attractant diacetyl and isoamyl alcohol, the volatile repellent 1-octanol, and the undefined cue Carolina mantle slug mucus, with ethanol as the negative control ( Figure 1A ). No significant differences in chemotaxis were observed between genotypes for any of the tested stimuli. To further evaluate responses to attractants, both strains were subsequently examined using diacetyl concentrations ranging from 1.14 × 10⁻⁵ M to 1.14 M ( Figure 1B ) and Carolina mantle slug mucus concentrations ranging from 1:0 to 1:100,000 v/v in ethanol (Figure 2C). Both genotypes exhibited similar concentration-dependent chemotaxis responses across the tested range, with no difference observed between wild-type and RB1526 animals. Taken together, these findings indicate that loss of *srd-44* does not significantly alter chemotaxis toward the tested volatile odorants or Carolina mantle slug mucus under the conditions examined, providing an opportunity for further investigation of its sensory function.

## Methods

N2 and RB1526 ( *srd-44 ( ok1831 )* ) strains were provided by the CGC, which is funded by NIH Office of Research Infrastructure Programs (P40 OD010440). The *srd-44 ( ok1831* ) allele contains a 2064 bp deletion withing the *srd-44* ( F17A2.8 ) locus on chromosome X and is predicted to cause transcript ablation of the primary *srd-44* transcript (Alliance of Genome Resources Consortium, 2024; Sternberg et al., 2024). This allele was generated by the *C. elegans* Gene Knockout Project at the Oklahoma Medical Research Foundation, which was part of the International *C. elegans* Gene Knockout Consortium. The *srd-44 ( ok1831* ) allele deletion was confirmed through a primer pair of inner left (cttgatcagtcgctctcgtg) and inner right (cgcaaccattttggagagac) following six backcrossings. *C. elegans* strains were maintained using standard nematode culture protocols as previously described (Stiernagle, 2006). Briefly, an OP50 *E. coli* lawn was grown on NGM-coated 60 mm petri plates. Picked eggs were transferred from freshly starved stock plates, incubated at 20°C for 2-3 days, then noted for presence of synchronized L4 and young adults. Worms were collected and pelleted using an M9 wash buffer before being transferred to a prepared testing plate.

Chemotaxis assay was adopted from Margie et al. (2013) by utilizing four quadrant divisions with two test and two control quadrants in a 10cm petri plate. 40-150 Pelleted worms were placed in the center and allowed to absorb into the agar surface for 5 minutes. Testing compounds, mixed with 0.5M sodium azide, were applied to designated areas. After one hour at room temperature, plates were placed into a 4°C cooler, then scored for worms that travelled 0.5 cm from the point of origin. Chemotaxis Index was calculated using the following equation: Chemotaxis Index = (# Worms in Both Test Quadrants - Worms in Both Control Quadrants) / (Total # of Scored Worms).

*Philomycus carolinianus* (Carolina Mantle slug) was collected from the deciduous forests surrounding Goucher College in Towson, Maryland USA. Slugs were placed in a terrarium for 24 hours before being transferred to a terrarium containing autoclaved dirt, rocks, and woody material. Slugs were fed a diet of autoclaved carrots and lettuce, misted with autoclaved tap water via a Honeywell humidifier, and allowed to acclimate for 3 days before mucus was collected. Cotton tipped applicators were used to collect mucus, then swirled in an Eppendorf tube with 45 µl of ethanol and stored in a 4°C cooler before being transferred to a -80°C freezer. Ethanol was selected as the solvent for mucus preparation as previous behavioral studies using chemically complex invertebrate samples have used ethanol and other solvents without detecting solvent dependent chemotactic responses (Archer et al., 2020). All slugs were returned to their native habitat at the end of the study.

Statistics were performed using Graphpad Prism software version 10.5.0 for windows. A two-way ANOVA was used to evaluate the effects of genotype and stimulus condition, followed by Tukey's multiple comparisons to assess genotype differences within each condition. Descriptive statistics were used to calculate mean and standard deviation. The figure was created using grouped summary data.

## Reagents

**Table:** 

Strain	Genotype	Available From
N2	*Caenorhabditis elegans*	CGC
RB1526	*srd-44 ( ok1831 ) *	CGC

**Table:** 

Species	Common Name	Location Acquired
*Philomycus carolinianus*	Carolina Mantle slug	Woods surrounding Goucher College

**Table:** 

Reagent	Source	Description
1-Octanol, ≥99% (GC)	Sigma Aldrich	Cat# 472328-100ML
Diacetyl, 98.0+%	TCI Chemicals	Cat# B0682-25ML
Ethanol, 200 proof	Pharmco	Cat# 111000190-1Gal
Isoamyl Alcohol, ≥98% (GC)	Sigma Aldrich	Cat# W205702-1KG-K

Statistical Analysis

**Table:** 

Tukey's multiple comparisons test (Wildtype vs. RB1526 )	Predicted (LS) Mean diff.	Adjusted P Value
Diacetyl	-0.2448 to 0.2867	0.8733
Carolina Mantleslug	-0.4895 to 0.1697	0.3300
Isoamyl Alcohol	-0.4793 to 0.06227	0.1265
1-Octanol	-0.07980 to 0.5368	0.1408
Ethanol	-0.3069 to 0.3522	0.8895
